# Glycine Protects against Hypoxic-Ischemic Brain Injury by Regulating Mitochondria-Mediated Autophagy via the AMPK Pathway

**DOI:** 10.1155/2019/4248529

**Published:** 2019-02-06

**Authors:** Chen-chen Cai, Jiang-hu Zhu, Li-xia Ye, Yuan-yuan Dai, Ming-chu Fang, Ying-ying Hu, Shu-lin Pan, Si Chen, Pei-jun Li, Xiao-qin Fu, Zhen-lang Lin

**Affiliations:** Department of Neonatology, The Second Affiliated Hospital and Yuying Children's Hospital of Wenzhou Medical University, Wenzhou, Zhejiang 325027, China

## Abstract

Hypoxic-ischemic encephalopathy (HIE) is detrimental to newborns and is associated with high mortality and poor prognosis. Thus, the primary aim of the present study was to determine whether glycine could (1) attenuate HIE injury in rats and hypoxic stress in PC12 cells and (2) downregulate mitochondria-mediated autophagy dependent on the adenosine monophosphate- (AMP-) activated protein kinase (AMPK) pathway. Experiments conducted using an *in vivo* HIE animal model and *in vitro* hypoxic stress to PC12 cells revealed that intense autophagy associated with mitochondrial function occurred during *in vivo* HIE injury and *in vitro* hypoxic stress. However, glycine treatment effectively attenuated mitochondria-mediated autophagy. Additionally, after identifying alterations in proteins within the AMPK pathway in rats and PC12 cells following glycine treatment, cyclosporin A (CsA) and 5-aminoimidazole-4-carboxamide-1-b-4-ribofuranoside (AICAR) were administered in these models and indicated that glycine protected against HIE and CoCl_2_ injury by downregulating mitochondria-mediated autophagy that was dependent on the AMPK pathway. Overall, glycine attenuated hypoxic-ischemic injury in neurons via reductions in mitochondria-mediated autophagy through the AMPK pathway both *in vitro* and *in vivo*.

## 1. Introduction

Hypoxic-ischemic injury in the neonatal brain is associated with detrimental and lethal consequences secondary to perinatal asphyxia [1]. Neonates diagnosed with hypoxic-ischemic encephalopathy (HIE) exhibit enhanced morbidity with poor clinical outcomes, which have increased awareness of this disorder worldwide [2]. In developed countries, approximately 1–8 per 1000 live births are diagnosed with HIE, while 26 per 1000 live births suffered from this serious disease in developing countries [3]. The high cost and complicated therapeutic strategies associated with HIE typically result in difficulties maintaining adherence to treatment for patients and families. Even worse, severe cases of HIE often result in poor prognoses that include lifelong issues, like cerebral palsy, epilepsy, mental retardation, learning and cognitive disabilities, and hearing loss [4]. Thus, the development of inexpensive and effective treatment modalities is necessary to allow families to continue beneficial treatments that enhance the long-term prognoses of neonates.

It has been shown that autophagy is highly correlated with the pathogenesis of HIE, although autophagy is an important degradation process designed to protect intracellular homeostasis under conditions of hypoxic-ischemic injury [5, 6]. To a large extent, this process also degrades a significant number of abnormal proteins, injured intracellular organelles, and degraded normal proteins to influence neuronal function under conditions where fewer organelles are working [7]. In particular, the selective degradation of mitochondria is an important process in the nervous system.

Mitochondria are organelles that supply energy to the cell and play important roles in the nervous system. Suffering from the hypoxic or ischemic condition, mitochondria are sensitive and becoming swelling or mitochondrial fusion [8]. Accumulating evidence suggests that mitochondrial dysfunction also plays a central role in a variety of nervous system diseases. For example, insults of hypoxic or ischemic conditions to neurons may result from generation of reactive oxygen species (ROS), particularly reactive oxygen-free radicals or other molecules, which act as crucial physiological components of intracellular signaling pathways [9]. However, most ROS are primarily generated by damaged mitochondria [10] that typically exhibit abnormalities in mitochondrial membrane potential (MMP). In parallel, MMP acts as an indicator of mitochondria-mediated autophagy [11]. For further investigation to study mitochondria-mediated autophagy among researches, cyclosporin A (CsA), as a traditional inhibitor, is widely used to suppress this autophagy process [12].

In addition to producing the majority of intracellular adenosine triphosphate (ATP), mitochondria are also the key factor to maintain the homeostasis within the intracellular environment. The adenosine monophosphate- (AMP-) activated protein kinase (AMPK) pathway is a key sensor of mitochondrial function and is sensitive to downward trends in ATP. Under conditions of energy imbalance or ROS generation in which AMPK signaling is disrupted, ATP levels decrease and intracellular AMP levels increase with particular subunits (*α*, *β*, and *γ*) of AMPK to activate the AMPK pathway. *α* subunit of AMPK acts as a catalytic role and regulatory *β* and *γ* subunits keep the stable condition to AMPK. Among these subunits, *γ* subunit contains nucleotide binding domains, while *β* subunit should be stimulated allosterically to activate AMPK. Furthermore, *α* subunit is pivotal to AMPK activation, including phosphorylation [13]. In our previous concepts, the AMPK pathway always benefits recovery and plays a vital role in the cellular environment. For example, the AMPK pathway exhibits a range of activation such that it protects neurons under conditions of hypoxic attack but does not benefit neurons under hypoxic conditions if it is overactivated [14]. In parallel, phosphorylation of AMPK did not always play a protective role to maintain the energy regeneration process. It may highly conserve autophagy kinase like Atg1, which mostly stimulates mitochondria-mediated autophagy [15]. Thus, the AMPK signaling pathway reflects the condition of mitochondrial function, such as fission or the recycling of the phospholipid membranes of mitochondria [16]. Furthermore, to figure out the relation between the AMPK pathway and mitochondria-mediated autophagy, a classical agonist of the AMPK pathway, 5-aminoimidazole-4-carboxamide-1-b-4-ribofuranoside (AICAR) [17], preserves AMPK activation *in vivo* and *in vitro*, which was confirmed in the present study.

Cobalt chloride (CoCl_2_), as a chemical compound, is widely acknowledged as a classical stimulator to hypoxic-ischemic condition, which triggers ROS generation and transcriptional dysfunction of some genes, including hypoxia-inducible factor 1 (HIF-1) and pCNA [18]. In particular, CoCl_2_-induced injury may destroy mitochondrial membrane potential, ATP level, and increase mitochondrial fission [19]. PC12 cell is a cell line derived from rat pheochromocytoma and its highly differentiated cell type is similar to neuronal characteristics, which is widely used as an *in vitro* model for observing neuronal pathogeneses [20]. Therefore, CoCl_2_-treated PC12 cell, as the hypoxic model, was used in our study to observe the mitochondria insults related with autophagy.

Glycine is a common substance present in numerous biomolecules where it plays a fundamental role in cellular metabolism. Glycine also acts as a neurotransmitter or N-methyl-D-aspartate receptor coagonist, which is reported for its effectiveness among neurodegenerative diseases, like Alzheimer's disease [21]. Moreover, mitochondria oxidatively decompose glycine into CO_2_, NH_4_^+^, NADH, and a methylene group to create methylenetetrahydrofolate, which sustains homeostasis [22]. However, the inhibition of oxidative phosphorylation and/or glycolytic energy production by glycine may protect intracellular energy production, which plays a protective role against neurological diseases, such as stroke or intracerebral hemorrhage [23]. Indeed, glycine protects against injuries mediated by hypoxia, hypoxia-reoxygenation, ROS, and chemical energy depletion [24]. Moreover, glycine has been increasingly employed as an effective therapeutic strategy for the protection of mitochondria in preclinical experiments in the liver [25] and kidney [26].

To date, few studies [27] have investigated whether glycine protects against hypoxic-ischemic injury in the brain by regulating mitochondria-mediated autophagy processes that are dependent on the AMPK pathway. Therefore, the present study is aimed at determining the role that glycine plays in neuroprotection via the regulation of mitochondria-mediated autophagy using both *in vivo* and *in vitro* models to aid future research.

## 2. Material and Methods

### 2.1. Drug and Reagents

Glycine (assay ≥ 98.5%; G5417-MSDS) and CoCl_2_·6H_2_O (C8661-25G) were purchased from Sigma-Aldrich (St. Louis, MO, USA). Dulbecco's modified Eagle medium (DMEM; 10569044) and fetal bovine serum (FBS; 10099141) were obtained from Gibco (Grand Island, NY, USA). 2,3,5-Triphenyltetrazolium chloride (TTC; 17779-10X10ML-F) was purchased from Sigma-Aldrich (St. Louis, MO, USA). 2′,7′-dichlorodihydrofluorescein diacetate (DCFH-DA; S0033) was purchased from Beyotime (Beijing, China). 5-Aminoimidazole-4-carboxamide-1-b-4-ribofuranoside (AICAR; A9978-25MG) was acquired from Sigma-Aldrich (St. Louis, MO, USA) and cyclosporin A (CsA; S2286) was obtained from Selleckchem (Houston, TX, USA). Cell counting kit-8 (CCK-8; CK04) was gained from Solarbio (Beijing, China). MitoSOX Red (40778ES50) was delivered from Yeasen (Shanghai, China). Tetramethylrhodamine ethyl ester perchlorate (TMRE; 87917-25MG) and Hoechst 33258 (94403-1ML) were also purchased from Sigma-Aldrich (St. Louis, MO, USA). 4,6-Diamidino-2-phenylindole (DAPI; C1002) and terminal deoxynucleotidyl transferase dUTP nick end labeling (TUNEL; C1088) were purchased from Beyotime (Shanghai, China).

Primary antibodies are as follows: mouse antihypoxia-inducible factor 1-alpha (HIF-1*α*, Abcam, Cambridge, MA, USA; H1alpha67), rabbit anti-Mitofusin 2 (Mfn-2, Cell Signaling Technology, Danvers, MA, USA; 9482s), mouse anti-parkin (Abcam, Cambridge, MA, USA; ab77924), rabbit anti-PINK1 (Abcam, Cambridge, MA, USA; ab23707), rabbit anti-p62 (Cell Signaling Technology, Danvers, MA, USA; 23214), rabbit anti-LC3 B (Cell Signaling Technology, Danvers, MA, USA; 3868), rabbit anti-Bnip3 (Abcam, Cambridge, MA, USA; ab109362), rabbit anti-Thr^172^ phosphorylated AMPK*_α_* (Cell Signaling Technology, Danvers, MA, USA; 50081), rabbit antiglyceraldehyde-3-phosphate dehydrogenase (GAPDH, Cell Signaling Technology, Danvers, MA, USA; 5174T), rabbit anti-AMPK*_α_* (Cell Signaling Technology, Danvers, MA, USA; 5832), and rabbit anti-Ser^2448^ phosphorylated mTOR (Cell Signaling Technology, Danvers, MA, USA; 5536). Secondary antibodies are as follows: goat anti-rabbit secondary antibody (ab150077) and rabbit anti-mouse secondary antibody (ab150125) were purchased from Abcam.

### 2.2. Animal Care, Surgery, and Ethical Certification

Sprague-Dawley (SD) rats were provided by the Animal Center of the Chinese Academy of Sciences, placed in individual housing of Wenzhou Medical University Animal Center (ethic number: wydw2014-0058), allowed free access to water and standard feed, and maintained in specific pathogen-free conditions under a 12/12 h light cycle at 23 ± 2°C and 60 ± 10% humidity. The rats were then crossbred to generate neonate SD rats that, on postnatal day 7 (P7), underwent minor surgery. Overall, 205 healthy neonatal 4-day-old rats were used in this study. 125 4-day-old rats were randomly allocated in 5 experimental groups and 25 4-day-old rats in each experimental group, and 80 7-day-old rats used to figure out the optimum dosage of glycine at a dose-dependent manner. For searching the optimum dosage of glycine, series of dose-dependent manner tests were used to figure out the most effective and economic dosage in order to gain the best recovery [28]. Briefly, 7-day-old pups in the HIE + glycine group were pretreated with an optimum intraperitoneal (i.p.) dose of glycine, while pups in the HIE + glycine + CsA and the HIE + glycine + AICAR groups were treated with CsA (5 mg/kg) [29] and AICAR (50 mg/kg) [30] started at 4-day-old rats, respectively, for 3 days, then followed by glycine administration at 7-day-old rats. The model employed in the present study was adapted from Vannucci and Vannucci [31]. For the surgery, the 7-day-old pups were anesthetized with anhydrous ether and underwent an incision at the middle of the neck followed by unilateral ligation of the left common carotid artery. Following suturing of the incision, the pups were allowed to recover for 2 h. Then, they were placed in a confined space with an environment consisting of humidified nitrogen-oxygen mixture (92% nitrogen and 8% oxygen, 3–4 l/min) for 2 h to induce further hypoxia. Pups in the sham group were anaesthetized, and the same midline incisions were performed without ligation of the common carotid arteries, followed with saline (the same volume, i.p.) pretreatment. This is aimed at guaranteeing the same surgical stimulations in both sham and experimental animals in order to exclude unrelated variables caused by surgery itself. All pups returned to their rearing cages for subsequent experimental procedures. In the following days, the rats were given daily administration of the drugs or saline. All animal procedures and operations were performed according to the animal use and care protocol based on the *Guide for the Care and Use of Laboratory Animals* from the National Institutes of Health and Wenzhou Medical University.

### 2.3. Evaluation of the Infarct Areas

To measure the infarct areas alleviated by drug administrations, 7-day-old rats from each group were anesthetized and sacrificed 24 h after surgery. 2,3,5-Triphenyltetrazolium chloride (TTC, Sigma-Aldrich, St. Louis, MO, USA) was used to quantify infarct volume and figure out the optimum dosage of glycine. Each brain was frozen at −20°C for 15 min and cut into 2 mm coronal slices. Next, the brain slices were immersed in a 1% TTC solution in the dark for 30 min at 37°C and incubated in 4% paraformaldehyde. Brain infarct areas were calculated using the ImageJ software (National Institutes of Health, Bethesda, MD, USA).

### 2.4. Measurement of Brain Water Content

To measure the dropsy status of injured hemispheres under hypoxic-ischemic condition and drug efficacy on the edema hemisphere, the 7-day-old rats were anesthetized with diethyl ether and sacrificed for brain analyses, as previously described [32]. The ipsilateral hemispheres from the sham group, HIE group, and HIE + glycine group were isolated to measure wet weight (accurate to 0.1 mg), and each hemisphere was placed in a drying oven (100°C) for 48 h to measure the dry weight (accurate to 0.1 mg) again. The percentages of brain water content in the hemisphere were calculated using the following equation: ([wet weight − dry weight]/wet weight) × 100%.

### 2.5. Histopathological Analysis

From the observation on pathological tissues, the isolated brains from each group among 7-day-old rats were collected 24 h after surgery and 14-day-old rats were gained 7 days after surgery or drug administration, perfused with phosphate-buffered saline (PBS; pH 7.4, 20 ml) followed by 4% (*w*/*v*) paraformaldehyde (20 ml). They were then embedded in paraffin and cut into coronal sections (5 *μ*m) for histological evaluation, which showed directly about integrity of hemisphere or functional neuron quantity between the cortex and hippocampus. The paraffin-embedded brain sections were dewaxed, rehydrated, and stained with hematoxylin and eosin (HE) or Nissl solution (Solarbio, Beijing, China). The results of the histological procedures were measured using light microscopy.

### 2.6. Transmission Electron Microscopy

To further understand the inner autophagy process or mitochondrial status among neurons, transmission electron microscopy (TEM, H-600IV; HITACHI, Tokyo, Japan) was used to observe directly this phenomenon. The 7-day-old pups were intracardially perfused with 2.5% glutaraldehyde and 2% paraformaldehyde in cacodylate buffer (0.1 mol/l, pH 7.4), and then the brains were postfixed overnight at 4°C in the same fixative. The brains were rinsed in 1% osmium tetroxide, dehydrated in an acetone series, infiltrated in Epon 812 for a longer period, and finally embedded. Next, ultrathin sections were cut with a diamond knife and stained with uranyl acetate and lead citrate. The sections were examined using TEM and the mitochondrial forms in both the cortex and hippocampus of each group were assessed.

### 2.7. Hindlimb Suspension Testing

After observing pathological alterations and subcellular structures above, the further neuronal functions or improvements should also be measured. Hindlimb suspension was performed to evaluate the right hindlimb muscular strength while its ipsilateral hemisphere was damaged. Among 14-day-old rats, a hindlimb suspension test [33] was used to detect neuromuscular function in the right hindlimb. Briefly, a jar that was padded at the bottom with medical cotton balls to mitigate the consequences of falling was used and hindlimbs of the rats were placed on the edge of a tube. Behavior was scored as follows: 4, normal hindlimb separation with the tail raised; 3, hindlimbs were relatively weak and close together but not touching each other; 2, hindlimbs were often touching; 1, apparent weakness of the hindlimbs with the tail raised and limbs in a clasped position; and 0, a constant clasped condition of the hindlimbs with the tail lowered or failure to hold onto the edge for any period of time.

### 2.8. Berderson Behavioral Test

The Berderson behavioral test [34] was employed to assess palsy in the contralateral limbs of the 28-day-old rats. Briefly, the rats were held gently by the tail, suspended one meter above a platform, and their forelimb flexion was observed and scored as follows: 0 (normal), rats extend forelimbs toward the floor with no neurological deficits; 1, the forelimb contralateral to the injured hemisphere was often flexed in different postures including slight wrist flexion, shoulder adduction with extension at the elbow, posturing with full flexion of wrist and/or elbow, and/or adduction with severe internal rotation of the shoulder; 2, rats were placed on a large paperboard that could be gripped by the claws, gentle lateral pressure was applied behind the shoulder until the forelimbs slid several inches (which was frequently shown in each direction with their tails held), and severely dysfunctional rats exhibited consistently reduced resistance to the lateral push toward the paretic side; and 3, rats consistently circled toward the paretic side. Each neurological examination was performed in a 3–5 min period using a double-blind procedure.

### 2.9. Longa Assessment

After 4 weeks after model surgery, neuronal function among 28-day-old rats is ideally mature among normal adult rats. Longa scores [35] are judged at 28-day-old rats; we observed each rat walking behaviors to evaluate its ipsilateral hemisphere injury or improvement by drug administration. Scores are as follows: 0, normal function, no neurologic deficits; 1, flexion of the right front paw observed while the tail was raised, mild neurological deficit; 2, spontaneous circling to the right when walking, moderate neurological deficit; 3, body slanted to the right when walking, severe neurological deficit; and 4, not able to walk spontaneously without possible loss of consciousness. Each neurological examination was performed three times using a double-blind procedure.

### 2.10. Cell Cultures and Treatment

Differentiated PC12 cells were provided by the Type Culture Collection of the Chinese Academy of Sciences (Shanghai, China). The cells were cultured in DMEM (Gibco, Grand Island, NY, USA) supplemented with 10% (*vol*/*vol*) FBS (Gibco, Grand Island, NY, USA) in a humidified incubator with an atmosphere of 5% CO_2_ at 37°C in 6-well plates (3 ml culture media per well) or 96-well plates (200 *μ*l culture media per well). In the CoCl_2_ group, the PC12 cells were only treated with CoCl_2_, whereas the PC12 cells in the CoCl_2_ + glycine group were pretreated with glycine (ps. 1 *μ*M glycine: 1 ml culture media added with 1 *μ*l 1 mM glycine, according to the dose-dependent manner) and then underwent hypoxic injury in the same manner as the CoCl_2_ group.

In another set of experiments, PC12 cells were pretreated with CsA (1 *μ*M, Selleckchem, Houston, TX, USA) for 24 h prior to the glycine and CoCl_2_ treatments. To further assess the functions of the AMPK pathway using *in vitro* experiments, PC12 cells were pretreated with AICAR (0.5 mM) for 3 h prior to the operations mentioned above.

### 2.11. Cell Viability Assay

Cell viability was measured with the nonradioactive cell counting kit-8 (CCK-8, Solarbio) to understand PC12 cell conditions (activation or survival). PC12 cells at a density of 1 × 10^4^ were seeded on a 96-well plate in DMEM supplemented with 10% (*vol*/*vol*) FBS and administered CoCl_2_ or glycine in a dose- and time-dependent manner after 12 h of cultivation. Next, a culture medium supplied with the CCK-8 solution was added for an additional hour and its absorbance was measured with a microplate reader (Tecan Group Ltd., Männedorf, Switzerland) at a wavelength of 490 nm.

### 2.12. Measurement of Intracellular ROS Production

To analyze the ROS generation under the hypoxic-ischemic environment, PC12 cells were digested with trypsin, washed twice with PBS, and finally incubated with the ROS-specific fluorescent probe dye dichlorodihydrofluorescein diacetate (DCFH-DA; Beyotime, Shanghai, China) for 30 min at 37°C. Following incubation, the cells were collected and suspended in washing buffer, and ROS production was detected using a flow cytometer.

### 2.13. Detection of Mitochondrial ROS

Mitochondrial ROS level was measured using MitoSOX Red (Yeasen, Shangai, China), which was a fluorescent indicator of mitochondrial superoxide anions. Briefly, PC12 cells were washed with Hank's balanced salt solution (HBSS) and incubated in the dark with 5 *μ*M MitoSOX Red for 20 min at 37°C. Additionally, cell nuclei were stained with 4′,6′-diamidino-2-phenylindol (DAPI, Beyotime) for 5 min at room temperature. The cells were washed with PBS and imaged using fluorescence microscopy (Olympus IX73) with excitation and emission filters of 510 and 580 nm, respectively.

### 2.14. Measurement of the MMP

The MMP was measured using confocal microscopy with tetramethylrhodamine ethyl ester (TMRE) staining (Sigma-Aldrich). This technique is aimed at discovering the function of mitochondria or indirectly reflecting the insults among mitochondria. Following administration of the study drugs as described above, the cells were treated with 50 nM TMRE and further incubated for 45 min at 37°C. Additionally, nuclei of the PC12 cells were loaded with Hoechst dye to determine the position of each cell.

### 2.15. Terminal Deoxynucleotidyl Transferase dUTP Nick End Labeling

Terminal deoxynucleotidyl transferase dUTP nick end labeling (TUNEL; Beyotime) assays were performed following the administrations of each drug. Subsequent to TUNEL staining, the cells were stained with a DAPI solution in the dark for 5 min at room temperature to identify nuclei. This tool helped to understand the apoptosis undergoing among cellular environments. Fluorescence imaging was performed with a fluorescence microscope (Olympus IX73), and the numbers of TUNEL-positive cells are presented as the percentage of positive nuclei/total nuclei × 100%.

### 2.16. Immunofluorescence Analysis

For the immunofluorescence analyses, PC12 cells were washed three times with PBS and incubated with 4% paraformaldehyde at 4°C for 25 min. Next, the cells were loaded with 0.3% Triton X-100 for 15 min and incubated in 5% bovine serum albumin (BSA) at 37°C for 30 min. The primary antibody anti-Mfn-2, an indicator of further figuring out the outer membrane protein of mitochondria and indirect reflection of mitochondrial situation, was applied to the cells at 4°C for 12 h, followed by incubation with an appropriate secondary antibody at 37°C for 1 h. Additionally, the nuclei were stained with DAPI; images from each group were obtained with a fluorescence microscope (Olympus IX73) and the data were analyzed with the ImageJ software.

### 2.17. Monodansylcadaverine Staining

Autophagic vacuoles are frequently observed during autophagy and can be visualized by monodansylcadaverine (MDC) staining [36]. In the present study, PC12 cells were seeded in six-well plates with sterile cover slips and each group of PC12 cells was incubated with 0.05 mM MDC (Sigma-Aldrich, USA) in culture medium in the dark for 30 min at 37°C following pretreatment with a drug or an operation. After the incubation, cells were washed with PBS and analyzed with a fluorescence microscope (Olympus IX73; Olympus, Tokyo, Japan).

### 2.18. Western Blot Analysis

For Western blot analyses, the cells were washed with PBS, loaded with radioimmunoprecipitation assay (RIPA) buffer with the PMSF protease inhibitor, and placed upon ice for 30 min. Tissue samples from the cortex and hippocampus were isolated from the ischemic hemisphere, and proteins were extracted using a mammalian tissue extraction reagent. Additionally, the protein samples were homogenized and analyzed as previously described [37]. Briefly, equivalent amounts of protein were separated by 10% sodium dodecyl sulfate polyacrylamide gel electrophoresis (SDS-PAGE), transferred to polyvinylidene difluoride (PVDF) membranes (0.22 *μ*m), and blocked with 5% skim milk for 2 h at room temperature. Subsequently, the membranes were incubated in the appropriate primary antibodies overnight at 4°C followed by incubation with a secondary antibody (at appropriate dilutions) for 2 h at room temperature. The membranes were quantitatively analyzed using the Image Lab software with densitometry after the addition of an enhanced chemiluminescence substrate (Bio-Rad, Hercules, CA, USA).

### 2.19. Statistical Analysis

All data are expressed as the mean ± standard error of the mean (SEM) and were analyzed with a one-way analysis of variance (ANOVA) test followed by Newman-Keuls tests for intergroup comparisons. *P* value less than 0.05 was considered to indicate statistical significance. *P* value < 0.01 indicated that the probability of the experimental value falling into the control distribution was lower than a *P* value < 0.05. *P* value < 0.001 indicated that the probability of the experimental value falling into the control distribution was lower than a *P* value < 0.01. All data were analyzed using the SPSS 17.0 software (SPSS Inc., Chicago, IL, USA).

## 3. Results

### 3.1. Glycine Attenuated Hypoxic-Ischemic Injury in the Brains of Neonatal Rats

**Figure 1:**
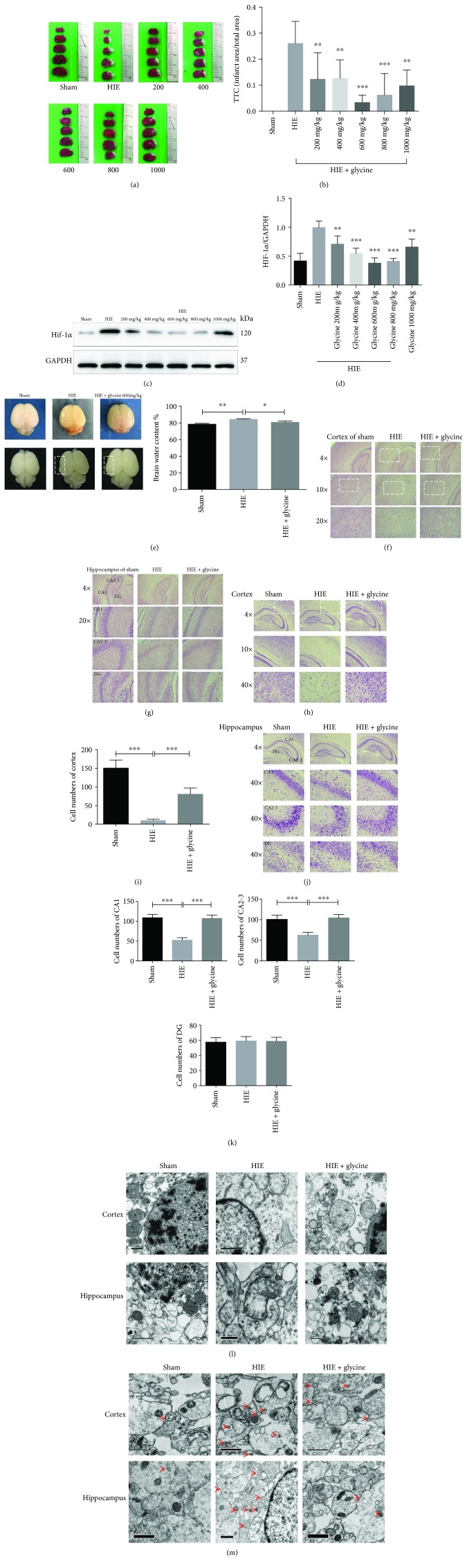
Glycine attenuated hypoxic-ischemic injury in the brains of neonatal rats. (a) TTC staining of sham rats, rats of HIE, and HIE rats with administration of glycine 200 mg/kg, 400 mg/kg, 600 mg/kg, 800 mg/kg, and 1000 mg/kg. (b) Calculation of infarct area shown by TTC staining (of the sham group). ^∗^ *P* < 0.05, ^∗∗^ *P* < 0.01, and ^∗∗∗^ *P* < 0.001 versus the HIE group. *n* = 5. (c) Protein expression level of HIF-1*α* of sham rats, rats of HIE, and HIE rats with administration of glycine 200 mg/kg, 400 mg/kg, 600 mg/kg, 800 mg/kg, and 1000 mg/kg. (d) Analyses of HIF-1*α* (of GAPDH). ^∗^ *P* < 0.05, ^∗∗^ *P* < 0.01, and ^∗∗∗^ *P* < 0.001 versus the HIE group. *n* = 3. (e) The brains were isolated from pups of the sham group, HIE group, and HIE with administration of the glycine group. Ratio of wet and dry is calculated in each group. *n* = 5. The brains (fixed with 4% paraformaldehyde) further showed dropsical areas and effectiveness of glycine administration. (f) HE staining of the cortex and hippocampus on the ipsilateral sides in rats of each group. (h) Nissl staining of the cortex on the ipsilateral sides in rats of each group is shown. (i) The analysis of cell number of neurons in the cortex on each group. ^∗∗∗^ *P* < 0.001. *n* = 5. (j) Nissl staining of the hippocampus on the ipsilateral sides in rats of each group. (k) Analysis of cell number of neurons in the hippocampus on each group. ^∗∗∗^ *P* < 0.001. *n* = 5. (l) Transmission electron microscope reveals status of mitochondria happened in tissues. (m) Transmission electron microscope discovers autophagosomes (red arrows) in tissues.

In the present study, infarct volume was quantified in rats from the sham, HIE, and HIE + glycine groups. In the HIE rats, glycine reduced the infarct area at an optimum dose of 600 mg/kg (dose/body weight) (Figures 1(a) and 1(b)). Western blot analyses assessing expression of the transcription factor hypoxia-inducible factor 1-alpha (HIF-1*α*) revealed that several different doses of glycine (200–1000 mg/kg) significantly reduced the expression of HIF-1*α*, indicating a rescue effect of glycine (Figures 1(c) and 1(d)). Therefore, all further experiments were performed with 600 mg/kg of glycine as the treatment standard. The rat brains of the sham, HIE, and HIE + glycine (600 mg/kg) groups were collected and their wet and dry ratios were measured. In the paraformaldehyde-fixed brains of each group, edema was easily identified in the left hemisphere of HIE rats but glycine administration attenuated the affected area to a certain extent (Figure 1(e)). Staining procedures in the cortex and hippocampus of the HIE group revealed more obvious edema than the sham group. However, these injuries were alleviated in the HIE + glycine group. Nissl stainings of the cortex and hippocampus were analyzed to assess neuronal viability (Figures 1(c)–1(k)). The number of functional neurons in Nissl-stained images was assessed to determine the effectiveness of glycine for treating or protecting neurons against hypoxic-ischemic injury. TEM images intuitively revealed the extent of mitochondrial injury in tissues. The HIE group exhibited swelling symptoms of injury in mitochondria, whereas the HIE + glycine group showed relatively normal mitochondria in neurons from the cortex and hippocampus (Figure 1(l)). Furthermore, the HIE group contained the highest quantity of autophagosomes, whereas the HIE + glycine group greatly reduced the quantity which exhibited the effectiveness of glycine upon alleviating autophagy.

### 3.2. Glycine Protected against Hypoxic-Ischemic Injury in the Brains via Attenuation of Mitochondrial-Mediated Autophagy

**Figure 2:**
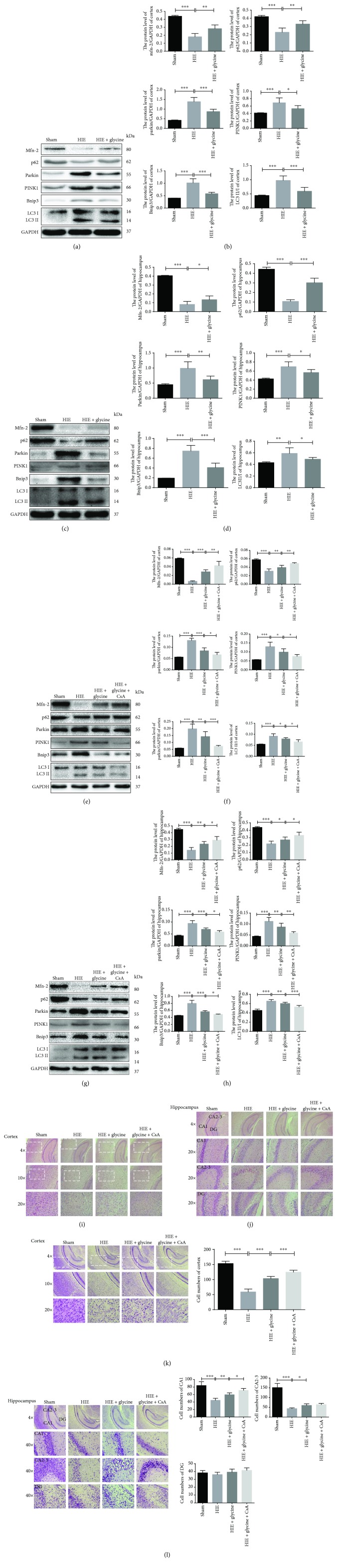
Glycine protected against hypoxic-ischemic injury in the brains via attenuation of mitochondrial-mediated autophagy. (a) Protein expression level of Mfn-2, p62, parkin, PINK1, Bnip3, LC3 II, and LC3 I on the cortex from ipsilateral sides of the sham group, HIE group, and HIE with administration of the glycine group (of GAPDH). (b) Analyses of Mfn-2, p62, parkin, PINK1, Bnip3, LC3 II, and LC3 I in each group. (c) Protein expression level of Mfn-2, p62, parkin, PINK1, Bnip3, LC3 II, and LC3 I on the hippocampus from ipsilateral sides of each group (of GAPDH). (e) Analyses of Mfn-2, p62, parkin, PINK1, Bnip3, LC3 II, and LC3 I in each group. (e) Protein expression level of Mfn-2, p62, parkin, PINK1, Bnip3, LC3 II, and LC3 I in each group from the cortex. (f) Analyses of Mfn-2, p62, parkin, PINK1, Bnip3, LC3 II, and LC3 I in each group. (g) Protein expression level of Mfn-2, p62, parkin, PINK1, Bnip3, LC3 II, and LC3 I in each group from the hippocampus. (h) Analyses of Mfn-2, p62, parkin, PINK1, Bnip3, LC3 II, and LC3 I in each group. (i) HE stainings of the cortex of four groups. (j) HE stainings of the hippocampus among four groups. (k) Nissl stainings in the cortex from four groups and neuron numbers were analyzed. ^∗∗∗^ *P* < 0.001. (l) Nissl stainings in the hippocampus from four groups and neuron numbers were analyzed. ^∗^ *P* < 0.05, ^∗∗^ *P* < 0.01, and ^∗∗∗^ *P* < 0.001. *n* = 5.

Following isolation of the cortex and hippocampus tissues, proteins were extracted for Western blot analyses. The protein levels of Binp3, p62, and microtubule-associated protein 1 light chain 3 (LC3 II/I) were assayed to determine the extent of autophagy. Additionally, Bnip3, parkin, PINK1, and Mfn2 protein levels were measured to evaluate how mitochondrial function mediated subsequent autophagy in the brain. The results indicated that protein expression in the cortex and hippocampus showed the same tendencies. The injured mitochondria exhibited dysfunction that was reflected by parkin and PINK1. Once dysfunction occurred within the mitochondria, PINK1 phosphorylated the parkin protein and both proteins accumulated on the outer membranes of mitochondria. Therefore, the expression of parkin and PINK1 was the highest among the HIE group, whereas Mfn-2 was expressed at a low level. Additionally, downstream autophagy-related proteins, such as Bnip3, parkin, PINK1, and LC3II/I, were significantly elevated in the tissues from the HIE group, whereas the HIE + glycine group showed significant improvements. Briefly, Bnip3 belongs to Bcl-2 family proteins, which acted as a sensitive factor in hypoxic condition [38] or mitochondrial dysfunction [39]. The previous study also indicated that upregulation of Bnip3 was related with the autophagy induction [40]. The level of LC3 was an important autophagic marker, which was widely used to identify autophagy proceed. p62 protein also played a role in autophagy that selectively decreased autophagy process [41]. Alteration of the protein expressions mentioned above revealed that glycine administration improved or recovered dysfunctional mitochondria and alleviated autophagy following hypoxic-ischemic injury. Following glycine treatment, parkin and PINK1 expression levels were lower than those of the HIE group and Mfn-2 protein expression began to increase. Similarly, the autophagic process was altered by glycine treatment and enhanced the recovery of mitochondria. Compared to the HIE group, p62 protein expressed at a higher degree while Bnip3 and LC3II/I protein levels were decreased a lot following glycine treatment (Figures 2(a)–2(d)).

To determine whether mitochondria-mediated autophagy played a role in the regulation of pathogenesis in the nervous system, rat neonates were pretreated with CsA beginning at P4 through the HIE operation and then received daily doses of glycine only. The cortical and hippocampal samples of the HIE + glycine + CsA group revealed a protective effect of glycine. Compared to the HIE + glycine group, Mfn-2 and p62 protein levels were enhanced by glycine, whereas parkin, PINK1, Bnip3, and LC3 II/I levels were lower, which illustrated that the brains exposed to hypoxic-ischemic injury exhibited extensive mitochondria-mediated autophagy and glycine attenuated these insults by regulating this autophagy (Figures 2(e)–2(h)). Moreover, histopathological analyses revealed that the cortex and hippocampus structures in the HIE + glycine + CsA group exhibited the best recovery among four groups (Figures 2(i) and 2(j)). Nissl staining procedures were conducted to determine neuron status among four groups and revealed that cortical neurons in the HIE + glycine + CsA group preserved a better functional status than in the HIE and HIE + glycine groups. In the hippocampus, neurons from CA1 and CA2–3 regions also exhibited good recovery (Figures 2(k) and 2(l)).

### 3.3. Glycine Eliminated Mitochondria-Mediated Autophagy via Regulation of the AMPK Pathway

**Figure 3:**
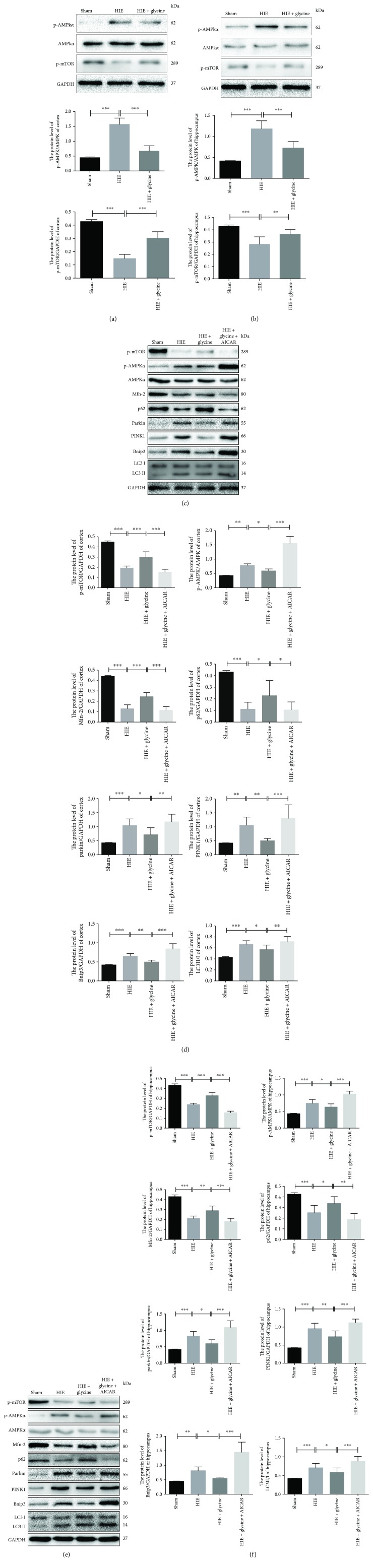
Glycine eliminated mitochondria-mediated autophagy via regulation of the AMPK pathway. (a) Protein expression level of p-AMPK*_α_* in the cortex from ipsilateral sides of each group and analysis of p-AMPK*_α_* (of AMPK*_α_*), p-mTOR (of GAPDH). (b) Protein expression level of p-AMPK*_α_* on the hippocampus from ipsilateral sides of each group and analysis. (c) Protein expressions of p-mTOR, p-AMPK*_α_*, AMPK*_α_*, Mfn-2, p62, parkin, PINK1, Bnip3, LC3 II, and LC3 I from the cortex. (d) Analyses of protein expressions above. (e) Protein expressions of p-mTOR, p-AMPK*_α_*, AMPK*_α_*, Mfn-2, p62, parkin, PINK1, Bnip3, LC3 II, and LC3 I from the cortex. (f) Analysis of protein expressions above. ^∗^ *P* < 0.05, ^∗∗^ *P* < 0.01, and ^∗∗∗^ *P* < 0.001. *n* = 5.

Proteins in the AMPK pathway, including p-AMPK*_α_* and p-mammalian target of rapamycin (p-mTOR), were also assessed and showed differences among groups in the cortex or hippocampus. p-AMPK*_α_* proteins were expressed at the highest level in the HIE group while p-mTOR expression decreased greatly. In the HIE + glycine group, glycine treatment alleviated the increase in p-AMPK*_α_* protein expression and improved p-mTOR expression, which confirmed that glycine administration influenced the AMPK pathway *in vivo* (Figures 3(a) and 3(b)).

To examine the relationships among HIE pathogenesis, glycine, and the AMPK pathway, an agonist of the AMPK pathway, AICAR, was employed to overactivate the AMPK pathway in rats under HIE operations. In the HIE + glycine + AICAR group, p-AMPK*_α_* protein expression increased, whereas p-mTOR protein expressions and other proteins associated with mitochondria-mediated autophagy exhibited poorer results than the HIE + glycine group. At the same time, parkin, PINK1, Bnip3, and LC3 II/I protein levels showed the highest degree, whereas Mfn-2 and p62 protein expressed the lowest level among four groups (Figures 3(c)–3(f)). Taken together, these results indicate that overactivation of AMPK blocked the effectiveness of glycine that downregulates the AMPK pathway and subsequently influences AMPK-regulated autophagy. In other words, compared with the HIE group, glycine administration could downregulate the AMPK pathway in the hypoxic-ischemic insult and alleviate mitochondria-mediated autophagy to gain the protection in the HIE + glycine group.

### 3.4. Glycine Improved Prognosis of Rats following Hypoxic-Ischemic Injury

**Figure 4:**
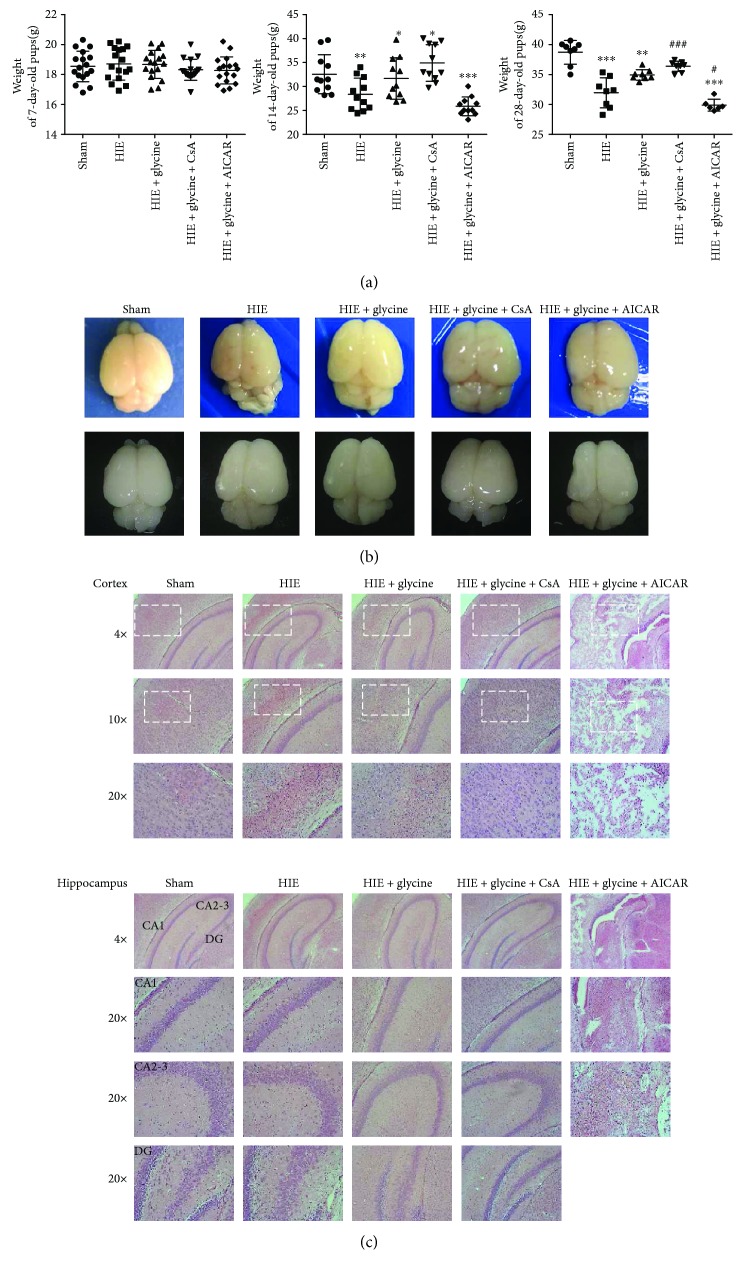
Glycine improved prognosis of rats following hypoxic-ischemic injury. (a) Weights of rats of 7 days, 14 days, and 28 days. ^∗^ *P* < 0.05 and ^∗∗∗^ *P* < 0.001. ^#^ *P* < 0.05, ^##^ *P* < 0.01, and ^###^ *P* < 0.001. (b) The brains isolated from 14-day-old rats of each group. Fresh brain tissue is shown at the first line and dehydrated brains tissue is shown at the second line. *n* = 3. (c) HE staining of the cortex and hippocampus from ipsilateral sides of each group. *n* = 3.

The bodyweights of the pups were assessed at three different ages: 7, 14, and 28 days. The bodyweights did not differ significantly in each group at 7 days (before operations). However, at 14 days, the HIE + glycine group showed improvements in bodyweight, compared with the HIE and HIE + glycine + AICAR groups. The HIE + glycine + CsA group exhibited the best weight gain among five groups, whereas rats from the HIE + glycine + AICAR group showed the lowest amount of weight gain. At 28 days, the rats of the HIE + glycine + AICAR group exhibited the lowest weight and two pups died. Following long-term glycine treatment, there was no significant difference between the HIE + glycine group and the HIE + glycine + CsA group, though these two groups exhibited better weight gains than the HIE group (Figure 4(a)).

At 7 days after surgery, three neonate rats from each group were sacrificed to further investigate the protective effects of glycine. Atrophy in the left brain showed significant improvements following treatment with glycine or glycine combined with CsA (Figure 4(b)). The HIE + glycine + AICAR group exhibited serious atrophy and had the worst prognosis among five groups. HE staining revealed the efficacy of glycine, especially in the HIE + glycine + AICAR group, in that the cortex and hippocampus exhibited considerable atrophy and were difficult to recognize. However, brain tissues from the HIE + glycine group and the HIE + glycine + CsA group exhibited better recovery than the HIE and HIE + glycine + ICAR groups (Figure 4(c)).

The present study also assessed neuromuscular ability in 14-day-old pups after surgery (Table 1). Over a period of 28 days, Longa and Berderson tests (Tables 2 and 3, respectively) were performed to measure neurodeficiencies in neuromuscular behaviors. The HIE + glycine + CsA group had the best scores on the behavioral test, whereas the condition of the HIE + glycine + AICAR group did not differ significantly from the HIE group (Table 1). At 28 days of age, the Longa test revealed that the HIE + glycine and HIE + glycine + CsA groups showed better mobility compared with the HIE and HIE + glycine + AICAR groups. Additionally, based on the results above, it was evident that the HIE + glycine + AICAR group did not perform well on the Longa test (Table 2). In parallel, the rats from this group also did not perform well on the Berderson test (Table 3).

### 3.5. Glycine Attenuated CoCl_2_-Induced Cytotoxicity, Cellular ROS, and Apoptosis in PC12 Cells

**Figure 5:**
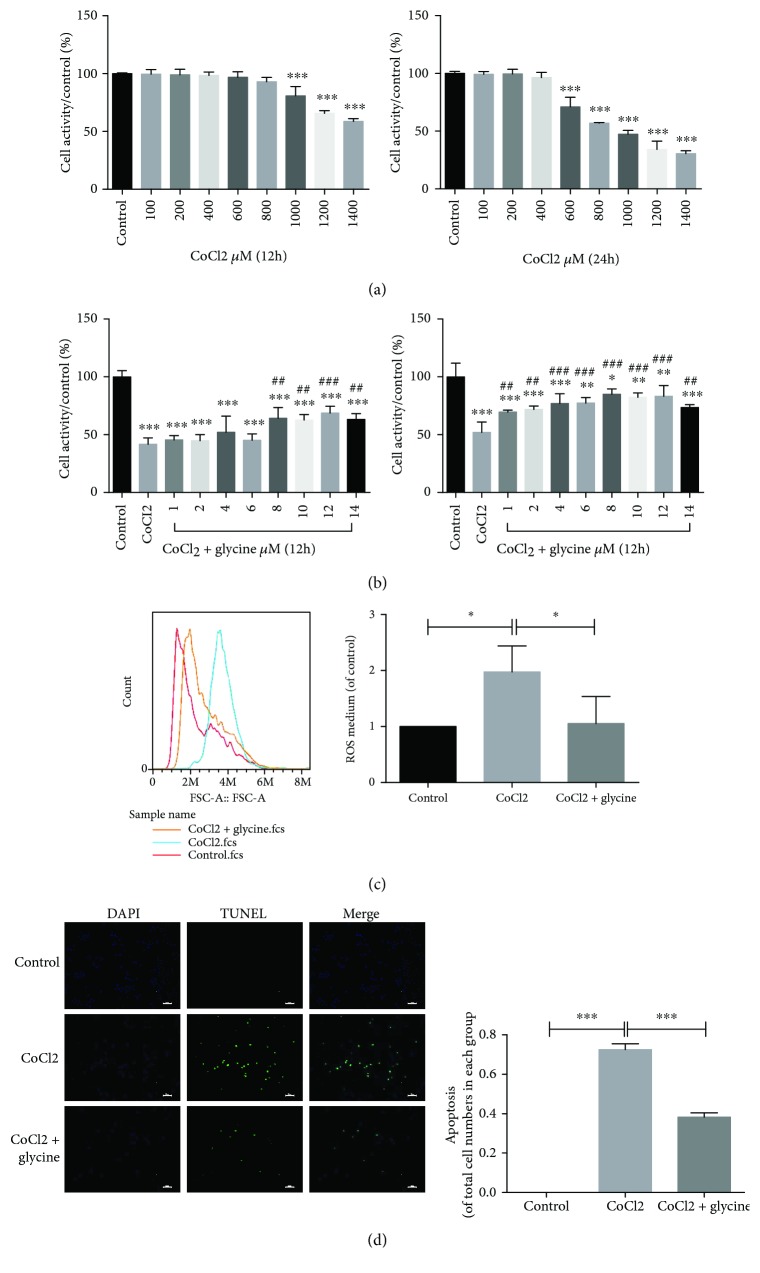
Glycine attenuated CoCl_2_-induced cytotoxicity, cellular ROS, and apoptosis in PC12 cells. (a) PC12 cells treated with CoCl_2_ on dose-dependent manner for 12 hours and 24 hours by CCK8. (b) PC12 cells were pretreated with different concentrations of glycine for 12 hours and 24 hours. Then, PC12 cells were treated with CoCl_2_ (800–1000 *μ*M) and cell viability was determined by CCK8. ^∗^ *P* < 0.05, ^∗∗^ *P* < 0.01, and ^∗∗∗^ *P* < 0.001 versus the control group. ^##^ *P* < 0.01 and ^###^ *P* < 0.001 versus the CoCl_2_ group. (c) ROS generation of the control group, CoCl_2_ group, and CoCl_2_ + glycine group was determined by flow cytometry assay of DCFH-DA. ^∗^ *P* < 0.05. *n* = 3. (d) Apoptotic cells of each group were detected by TUNEL and DAPI staining. ^∗∗∗^ *P* < 0.001. *n* = 3.

The initial effects of CoCl_2_ on neurons were detected using the CCK8 method. In the present study, CoCl_2_ (800–1000 *μ*M) significantly inhibited cellular viability within 24 h in a dose- and time-dependent manner compared with the control group (Figure 5(a)). Moreover, the optimum dose of glycine (8–10 *μ*M) effectively protected PC12 cells against CoCl_2_-induced injury (800–1000 *μ*M) in the same manner as the CoCl_2_ group (Figure 5(b)). DCFH-DA fluorescence was used to detect the level of ROS generation in each group and revealed that glycine pretreatment decreased ROS generation and protected against CoCl_2_-induced injury in PC12 cells (Figure 5(c)). TUNEL staining indicated that glycine also downregulated apoptosis to protect PC12 cells (Figure 5(d)).

### 3.6. Glycine Attenuated CoCl_2_-Induced Mitochondrial ROS Generation, Restored the MPP, Improved Mfn-2 Protein Levels, and Alleviated Autophagy in PC12 Cells

**Figure 6:**
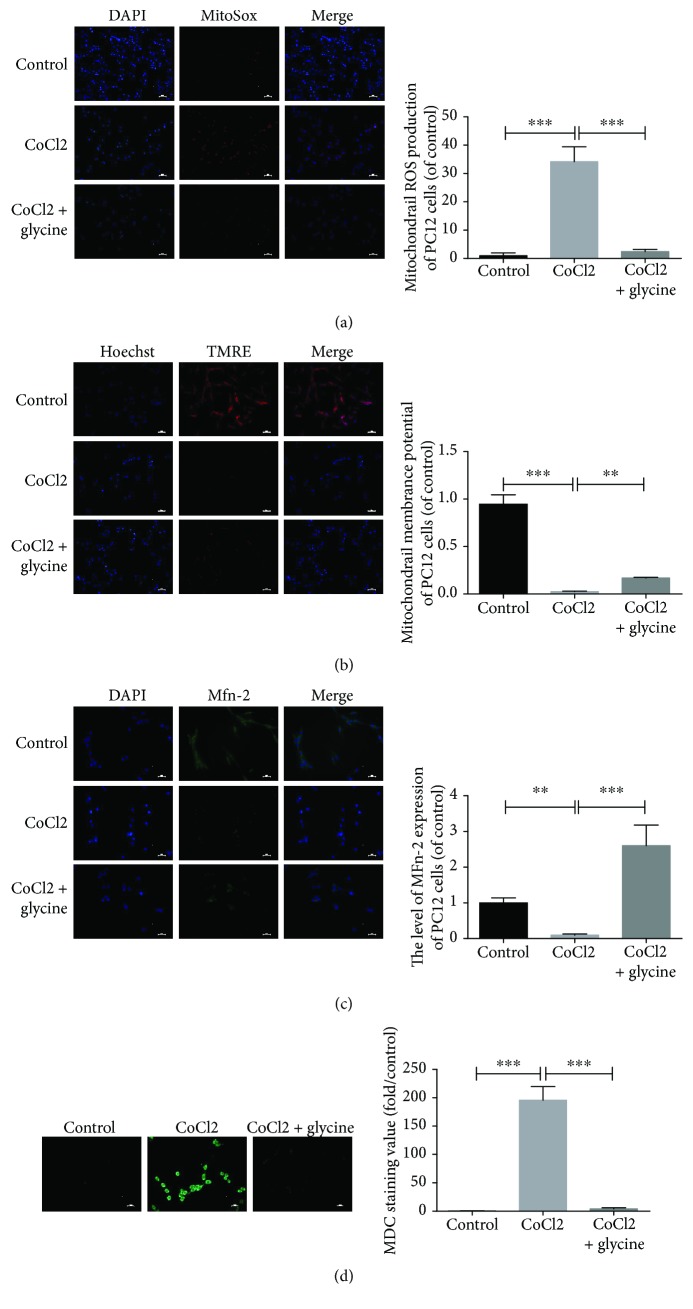
Glycine attenuated CoCl_2_-induced mitochondrial ROS generation, restored the MPP, improved Mfn-2 protein levels, and alleviated autophagy in PC12 cells. (a) Mitochondrial ROS generation of each group was detected by MitoSOX and DAPI staining. (b) Mitochondrial membrane potential of each group was determined by TMRE and Hoechst staining. (c) Protein expression level of Mfn-2 from outer membrane of mitochondria was measured by immunofluorescence. (d) Autophagic vacuoles from each group were measured by MDC staining. ^∗∗^ *P* < 0.01 and ^∗∗∗^ *P* < 0.001. *n* = 3.

As an indication of injured status in cells, mitochondria largely generated superoxide molecules by influencing various signaling pathways. MitoSOX, which confirmed the position of the mitochondrial matrix and detected superoxide anions in mitochondria, was used to assess oxidative stress in mitochondria of PC12 cells. Cells that underwent CoCl_2_ injury showed a significant increase in oxidative stress in mitochondria (Figure 6(a)), but the administration of an optimum dose of glycine lowered ROS generation. Additionally, PC12 cells were loaded with TMRE to evaluate the MMP, which reflected mitochondrial function. CoCl_2_ impaired mitochondria by decreasing the MMP, but glycine dramatically promoted the MMP (Figure 6(b)). Furthermore, Mfn-2 expression was at a low level in the CoCl_2_ group (Figure 6(c)), but glycine upregulated the Mfn-2 protein expression. According to the results of MDC staining, which specifically identified autophagic vacuoles, showed that the CoCl_2_ + glycine group gained the lower value of MDC immunofluorescence than the CoCl_2_ group. This result also illustrated that glycine could downregulate autophagy (Figure 6(d)).

### 3.7. Glycine Protected PC12 Cells against CoCl_2_-Induced Injury via Regulation of Mitochondria-Mediated Autophagy

**Figure 7:**
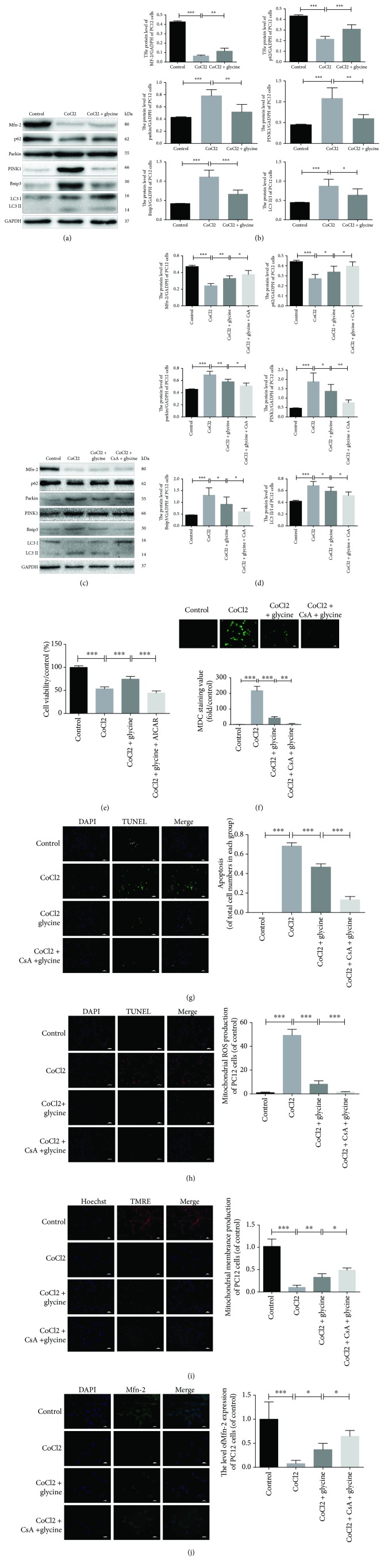
Glycine protected PC12 cells against CoCl_2_-induced injury via regulation of mitochondria-mediated autophagy. (a) Protein expression level of Mfn-2, p62, parkin, PINK1, Bnip3, LC3 II, and LC3 I in PC12 cells of each group. (b) Analyses of Mfn-2, p62, parkin, PINK1, Bnip3, LC3 II, and LC3 I in each group. (c) After treatment of CsA, protein expression levels of Mfn-2, p62, parkin, PINK1, Bnip3, LC3 II, and LC3 I in PC12 cells of four groups were determined by Western blotting. (d) Analysis of all protein expressions. (e) Cell viability of four groups was detected by CCK8. (f) Apoptotic cells from each group were measured by TUNEL and DAPI staining. (g) Mitochondrial ROS generations were detected by immunofluorescence of MitoSOX and DAPI staining. (h) Mitochondrial membrane potential was detected in each group by TMRE and Hoechst staining. (i) The Mfn-2, protein of mitochondrial outer membrane, was used to measure function of mitochondria. ^∗^ *P* < 0.05, ^∗∗^ *P* < 0.01, and ^∗∗∗^ *P* < 0.001. *n* = 3.

The present study examined the expression of parkin, PINK1, Mfn-2, Bnip3, p62, and LC3II/I (Figures 7(a) and 7(b)) and found increased expression of parkin, PINK1, Bnip3, and LC3II/I in the CoCl_2_ group. However, glycine pretreatment before CoCl_2_ (CoCl_2_ + glycine) significantly reduced such effect. At the same time, the expressions of Mfn-2 and p62 decreased following CoCl_2_ injury, but glycine pretreatment promoted the expressions of Mfn-2 and p62. CsA, a conventional inhibitor of mitochondria-mediated autophagy, was used to determine whether glycine regulated autophagy. Following treatment with a combination of CsA and glycine, the expressions of parkin, PINK1, Bnip3, and LC3 II/I were largely inhibited, whereas the expression of Mfn-2 and p62 was increased (Figures 7(c) and 7(d)). Additionally, the administration of CsA increased cellular viability such that it was better compared with the CoCl_2_ + glycine group (Figure 7(e)).

MDC staining revealed that the CoCl_2_ + glycine + CsA and CoCl_2_ + glycine groups exhibited a less degree of autophagy, particularly the CoCl_2_ + glycine + CsA group (Figure 7(f)). TUNEL staining in PC12 cells further confirmed that glycine protected PC12 cells (Figure 7(g)). Assessments of mitochondrial function included measurements of mitochondrial ROS generation, MMP, and Mfn-2 expression (Figures 7(h)–7(j)). In all four groups, glycine significantly decreased mitochondria-mediated autophagy, especially via the parkin/ PINK1 pathway, which has been linked to mitochondria-mediated autophagy.

### 3.8. Glycine Protected PC12 Cells against CoCl_2_ Injury via Regulation of AMPK-Dependent Mitochondria-Mediated Autophagy

**Figure 8:**
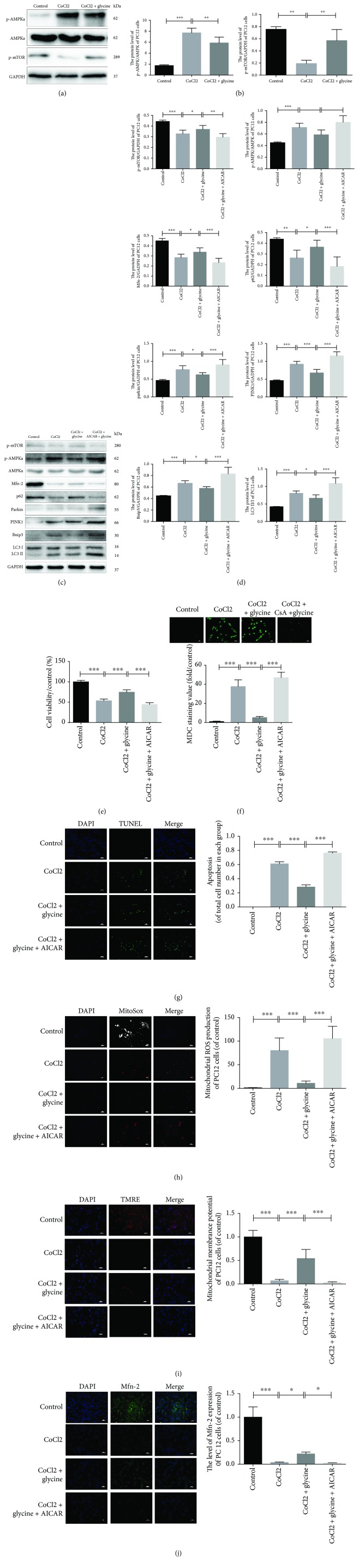
Glycine protected PC12 cells against CoCl_2_ injury via regulation of AMPK-dependent mitochondria-mediated autophagy. (a) p-AMPK*_α_* protein expression was detected by Western blotting in each group. (b) Analyses of p-AMPK*_α_* protein (of AMPK*_α_*) and p-mTOR (of GAPDH). (c) Agonist of the AMPK pathway, CsA, was used to overactivate proteins from the AMPK pathway. Protein expressions of four groups were detected by Western blotting. (d) Analysis of all protein expressions. (e) Cell viability in four groups was also measured by CCK8. (f) Autophagic vacuoles from each group were measured by MDC staining. (g) Apoptotic cells from each group were measured by TUNEL and DAPI staining. (h) Mitochondrial ROS generations were detected by immunofluorescence of MitoSOX and DAPI staining. (i) Mitochondrial membrane potential was detected in each group by TMRE and Hoechst staining. (j) Mfn-2, protein of mitochondrial outer membrane, was used to measure function of mitochondria. ^∗^ *P* < 0.05, ^∗∗^ *P* < 0.01, and ^∗∗∗^ *P* < 0.001. *n* = 3.

Because AMPK is phosphorylated upon activation of the AMPK pathway, it acts an energy sensor that reflects the condition of mitochondria. Analyses of p-AMPK expression in PC12 cells revealed high levels of p-AMPK*_α_* in the CoCl_2_-treated group, but glycine pretreatment reduced the levels of p-AMPK*_α_*. The mTOR pathway is the downstream of the AMPK pathway, and assessments in the present study showed that CoCl_2_ injury had an adverse effect on this pathway as well (Figures 8(a) and 8(b)). To determine whether the AMPK pathway modulated CoCl_2_-induced mitochondria-mediated autophagy, AICAR, an AMPK pathway agonist was used to modulate protein expression. In the CoCl2 + glycine + AICAR group, the expressions of parkin, PINK1, Bnip3, LC3II/I, and p-AMPK*_α_* were evident at a high degree while the expressions of Mfn-2, p-mTOR, and p62 were low (Figures 8(c) and 8(d)). At the same time, the group administrated CsA in combination with glycine exhibited the lowest viability (Figure 8(e)).

MDC staining revealed that the CoCl_2_ + glycine + AICAR group had the highest intensity of autophagy of the four groups, whereas the CoCl_2_ + glycine group exhibited attenuations in autophagy (Figure 8(f)). Moreover, TUNEL staining revealed that glycine pretreatment protected against CoCl_2_ injury, whereas overactivation of the AMPK pathway in the CoCl_2_ + glycine + AICAR group produced the highest number of TUNEL-positive cells (Figure 8(g)). Immunofluorescence analyses of the PC12 cells revealed that the mitochondrial function of the CoCl_2_ + glycine + AICAR group did not show significant improvements with the CoCl_2_ group (Figures 8(h)–8(j)). However, the CoCl_2_ + glycine group exhibited high levels of protection against CoCl_2_ injury. Parallel analyses confirmed the overactivation of the AMPK pathway by AICAR. This result suggests that the AMPK pathway played an important role in CoCl_2_-induced mitochondria-mediated autophagy.

## 4. Discussion

The present study was the first to investigate whether glycine could protect against hypoxic-ischemic injury in the nervous system by regulating mitochondria-mediated autophagy via the AMPK pathway using *in vivo* and *in vitro* experiments.

Pathogeneses of HIE have been studied a lot, such as endoplasmic reticulum stress [42], inflammation among white matter [43], or neuron apoptosis [44]. In recent years, autophagy has become the new target in various systems. Without exception, a few researches illustrated the relation between HIE and autophagy. Previous studies have demonstrated that autophagy is related to a wide variety of physiological and pathological processes [45, 46]. Particularly in hypoxic-ischemic situations, autophagy plays a vital role in the metabolic regulation of different organs [47–49]. Furthermore, autophagy represents a significant mechanism by which cellular homeostasis is maintained to allow for further adjustments in HIE [50, 51]. Compared with some results about autophagy, their opinions about appropriately increasing profitable autophagy gained the protection in HIE. It is true that autophagy gains two extremes, protection and destruction [52]. An excessive degree of autophagy can be harmful to the whole cell or tissues [53, 54], which was demonstrated in the present study. Our results demonstrated that the injured side of the brain was severely hydrophobic, exhibited signs of cortical liquidation, and showed destruction of the cortex and hippocampus following HIE operations. Additionally, there was a high level of LC3 protein expression, which is a traditional indicator of autophagy in the HIE and CoCl_2_ groups. Bnip3, which can be used to measure autophagy, was also expressed at a high level in the HIE and CoCl_2_ groups. This is similar to the findings of previous studies showing that Bnip3 is sensitive to hypoxic-ischemic pathogenesis and may potentially serve as a biomarker of excessive autophagy, which has been verified in other studies [55]. CsA, a conventional inhibitor of mitochondria-mediated autophagy, largely decreased the intensity of autophagy compared with the CoCl_2_ + glycine and HIE + glycine groups. In the *in vivo* experiments, neonatal rats were pretreated with CsA to inhibit autophagy and brain samples from these animals showed good recovery when CsA was combined with daily glycine administration. Similar results were obtained from the *in vitro* experiments that revealed good cell viability and MMP. Taken together, the present findings indicate that high levels of mitochondria-mediated autophagy degraded abnormal proteins and digested organelles, particularly mitochondria but influenced normal functioning proteins and organelles, which could be catastrophic to the cellular environment and body. The present TEM results easily identified edematous mitochondria in all three groups of rats. Outer membrane protein levels were also assessed to determine mitochondrial function. The expression levels of Mfn-2 are indicative of the dysfunction or fusion of mitochondria, which, in turn, activates autophagy [56, 57]. Moreover, PINK1 phosphorylates parkin and recruits it to the mitochondria, which damage these organelles. In the HIE group and CoCl_2_ group, the high expression levels of parkin and PINK1 indicated serious damage to the mitochondria. However, MDC staining in PC12 cells revealed that glycine treatment effectively alleviated the autophagy process compared with the CoCl_2_ and CoCl_2_ + glycine + AICAR groups. Therefore, novel treatments that immediately downregulate mitochondria-mediated autophagy are urgently needed to aid adjustment and recovery.

The administration of glycine is an effective treatment following hypoxic or hypoxic-ischemic injury in the liver [58], kidney [26], and various cell types [59–61]. In the present study, there were significant decreases in LC3 and Bnip3 protein levels following glycine pretreatment. At the same time, electron microscopy analyses indicated that glycine inhibited excessive autophagy. It is known that glycine protects against energy depletion by promoting the metabolism of intracellular or mitochondrial functions [24]. In the present study, glycine attenuated hypoxic-ischemic injury in the brain and improved mitochondrial function. HE and Nissl staining procedures revealed that glycine decreased the number of dying neurons and ameliorated the expression of related proteins while immunofluorescent analyses showed that outer membrane proteins in the mitochondria were altered by glycine. Taken together, these findings indicated that glycine could be an effective strategy for the promotion of mitochondrial function. Furthermore, glycine downregulated proteins related to mitochondria-mediated autophagy. Moreover, seven consecutive days of treatment with glycine resulted in a good prognosis in the behavioral test in 28-day-old rats. However, it has also been shown that glycine regulates the basic states of function in astrocytes and microglia by activating glycine receptors to promote recovery in the nervous system [62]. The present study did not verify the effectiveness of glycine in nonneuronal cell types.

AMPK is an energy-sensing center activated by stress or dysfunction in mitochondria [16, 63]. ATP deficiencies, or an increased ratio of AMP to ATP, usually originate from dysfunction of mitochondria. Under the hypoxic-ischemic insult, the mitochondria are sensitive (observing mitochondrial status), generating ROS and making chaos in energy supply. Subsequently, activation of the AMPK pathway (activated by *α* subunit in the ATP/AMP disorder) modulates mitochondrial function under conditions of stress, hypoxia, and/or ischemia [64]. In this process, extensive phosphorylation of AMPK also maintains autophagy kinase, which mostly induces mitochondria-mediated autophagy. Analyzing autophagy protein expressions among animal and cell models, we will help to directly understand the condition of mitochondria-mediated autophagy *in vivo* and *in vitro*.

Previous studies have demonstrated that mTOR protein expression is inhibited by activation of AMPK and that mTORC2 [65], as part of mTOR, plays a role to participate in the autophagy process [66]. Under hypoxic attack and other unfavorable conditions, AMPK activation inhibits the mTOR signaling pathway. In the present study, there were high levels of phosphorylation of AMPK expressed but low levels of p-mTOR following the HIE group or CoCl_2_ injury.

One previous study reported that the suppression of AMPK activity had no harmful effects on the basic functions of mitochondria and good recovery of cellular metabolism was evident on following treatment [67]. However, the role of the AMPK pathway is complex and may vary under conditions. Within a specific range, AMPK plays a protective role; however, overactivation of the AMPK pathway can worsen intracellular status, especially in neurons exposed to a hypoxic environment [14]. To further understand the relationship of glycine with the AMPK pathway, AICAR, an agonist of AMPK, was administrated to measure autophagic proteins. Glycine treatment inhibited AICAR-induced activation of the AMPK pathway and downregulated autophagy. Of the three groups in the present study, p-AMPK expression levels were highest following HIE or CoCl_2_ injury. However, the administration of AICAR in combination with glycine demonstrated the activation of AMPK proteins, ROS degeneration, and worsened mitochondrial function, which then enhanced autophagy. Detailed molecular mechanisms underlying inactivation of the AMPK pathway by glycine still require further investigation. However, previous studies have suggested several possibilities [68]. For example, fatty acid oxidation increases and glucose oxidation suppressed following AMPK activation [69, 70]. However, glycine pretreatment may attenuate fatty acid oxidation and adjust glucose oxidation to normal levels, which would aid in recovery of the whole neuron and its functions. AICAR, as an agonist of the AMPK pathway, revealed that activation of the AMPK pathway had detrimental effects. Rats that received daily treatments of AICAR and glycine did not show good recovery in terms of protection or further treatment. Similarly, PC12 cells pretreated with AICAR and glycine exhibited poor recovery following overactivation of the AMPK pathway.

There are several limitations of the present study that should be noted when interpreting the results. HIE models typically use knockout rats to assess the manner in which glycine ameliorates HIE injury. Moreover, primary neurons can be collected and analyzed to assess the effects of glycine but evaluation of various cell types would strengthen the present results. Finally, it remains to be elucidated whether glycine regulates fatty acid oxidation.

In summary, the present data provide the first evidence that glycine attenuated hypoxic-ischemic injury in neurons or the nervous system by decreasing mitochondria-mediated autophagy through regulating the AMPK pathway using both in vitro and in vivo experiments. Since the pathogeneses of HIE are currently too complex to develop accurate and effective therapy, glycine may serve as a more inexpensive and effective alternative to other medicines. Additionally, identifying the optimum dosage of glycine for improved prognosis will be important.

## Figures and Tables

**Table 1:** Assessment of hindlimb suspension of 14-day-old pups.

Group	Number	Hindlimb suspension
Sham	11	4.00 ± 0.00
HIE	11	1.24 ± 0.11^∗∗∗^
HIE + glycine	11	2.15 ± 0.12^∗∗∗^^,###^
HIE + glycine + CsA	11	2.60 ± 0.11^∗∗∗^^,###,&&^
HIE + glycine + AICAR	11	1.30 ± 0.11^∗∗∗^^,&&&^

Dates were presented as mean ± SEM from rats from each group. ^∗∗∗^ *P* < 0.001 versus the sham group. ^###^ *P* < 0.001 versus the HIE group. ^&&^ *P* < 0.01 and ^&&&^ *P* < 0.001 versus the HIE + glycine group.

**Table 2:** Longa scores of 28-day-old pups.

Group	Number	Hindlimb suspension
Sham	8	0.00 ± 0.00
HIE	8	1.75 ± 0.14^∗∗∗^
HIE + glycine	8	1.00 ± 0.13^∗∗∗^^,###^
HIE + glycine + CsA	8	0.83 ± 0.13^∗∗∗^^,###^
HIE + glycine + AICAR	6	1.70 ± 0.13^∗∗∗^^,&&&^

Dates were presented as mean ± SEM from rats from each group. ^∗∗∗^ *P* < 0.001 versus the sham group. ^###^ *P* < 0.001 versus the HIE group. ^&&&^ *P* < 0.001 versus the HIE + glycine group.

**Table 3:** Berderson assessment of 28-day-old pups.

Group	Number	Hindlimb suspension
Sham	8	0.00 ± 0.00
HIE	8	2.08 ± 0.15^∗∗∗^
HIE + glycine	8	1.45 ± 0.10^∗∗∗^^,###^
HIE + glycine + CsA	8	0.95 ± 0.11^∗∗∗^^,###,&&^
HIE + glycine + AICAR	6	2.04 ± 0.14^∗∗∗^^,&&&^

Dates were presented as mean ± SEM from rats from each group. ^∗∗∗^ *P* < 0.001 versus the sham group. ^###^ *P* < 0.001 versus the HIE group. ^&&^ *P* < 0.01 and ^&&&^ *P* < 0.001 versus the HIE + glycine group.

## Data Availability

The data used to support the findings of this study are available from the corresponding author upon request.

## Supplementary Materials

Supplementary Materials Supplementary Figure 1. The dosage of glycine pretreatment has no effect on normal cortex status of neonatal rats. (A) Protein expressions were examined for evaluating mitochondria-mediated autophagy extent between the sham and sham + glycine groups. (B) Analyses of all protein expressions. *n* = 5. Supplementary Figure 2. The dosage of glycine pretreatment has no effect on normal hippocampus status of neonatal rats. (A) Protein expressions were examined for evaluating mitochondria-mediated autophagy extent between the sham and sham + glycine groups. (B) Analyses of all protein expressions. *n* = 5. Supplementary Figure 3. The dosage of glycine administration has no influence on normal status of PC12 cells. (A) Protein expressions were examined for evaluating mitochondria-mediated autophagy extent between control and control + glycine groups. (B) Analyses of all protein expressions. *n* = 3. (C) Cell viability between two groups. *n* = 5.
