# Supplementary material for: Glycine Protects against Hypoxic-Ischemic Brain Injury by Regulating Mitochondria-Mediated Autophagy via the AMPK Pathway
Source: Oxid Med Cell Longev. 2019 Feb 6;2019:4248529. doi: 10.1155/2019/4248529 (PMC6381570; doi:10.1155/2019/4248529)
Supplement: Supplementary Materials — Supplementary Figure 1. The dosage of glycine pretreatment has no effect on normal cortex status of neonatal rats. (A) Protein expressions were examined for evaluating mitochondria-mediated autophagy extent between the sham and sham + glycine groups. (B) Analyses of all protein expressions. n = 5. Supplementary Figure 2. The dosage of glycine pretreatment has no effect on normal hippocampus status of neonatal rats. (A) Protein expressions were examined for evaluating mitochondria-mediated autophagy extent between the sham and sham + glycine groups. (B) Analyses of all protein expressions. n = 5. Supplementary Figure 3. The dosage of glycine administration has no influence on normal status of PC12 cells. (A) Protein expressions were examined for evaluating mitochondria-mediated autophagy extent between control and control + glycine groups. (B) Analyses of all protein expressions. n = 3. (C) Cell viability between two groups. n = 5. [file 4248529.f1.pdf]

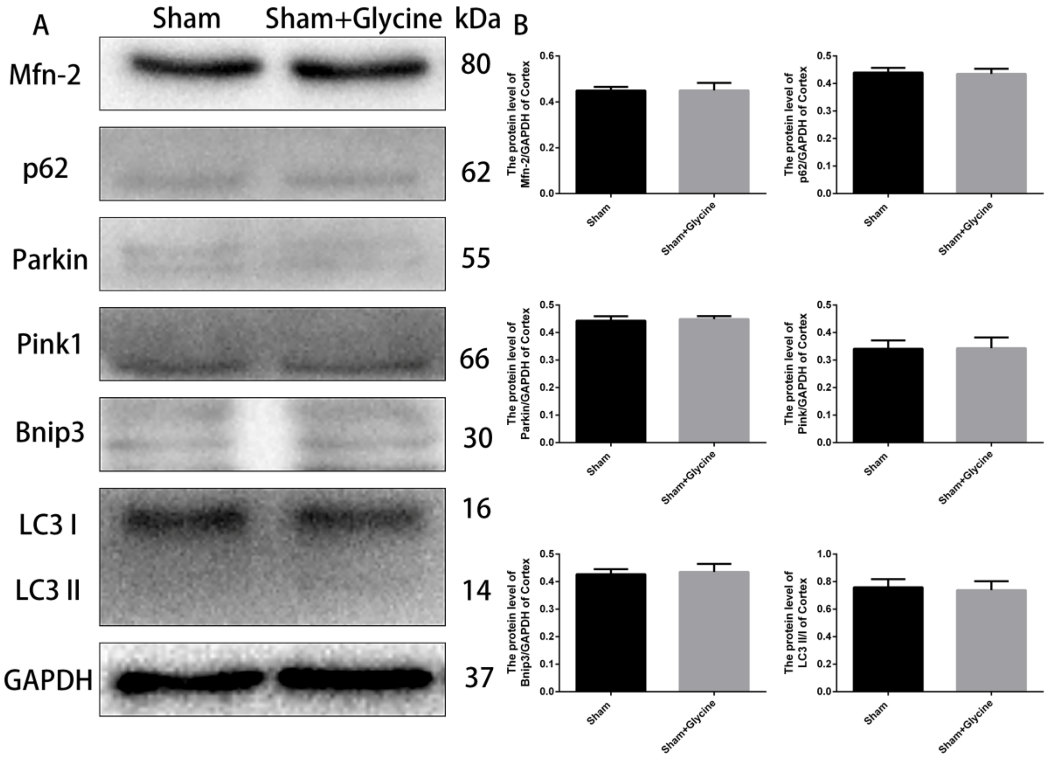

Suppl figure 1. The dosage of glycine pretreatment has no effect on normal cortex status of neontal rats.

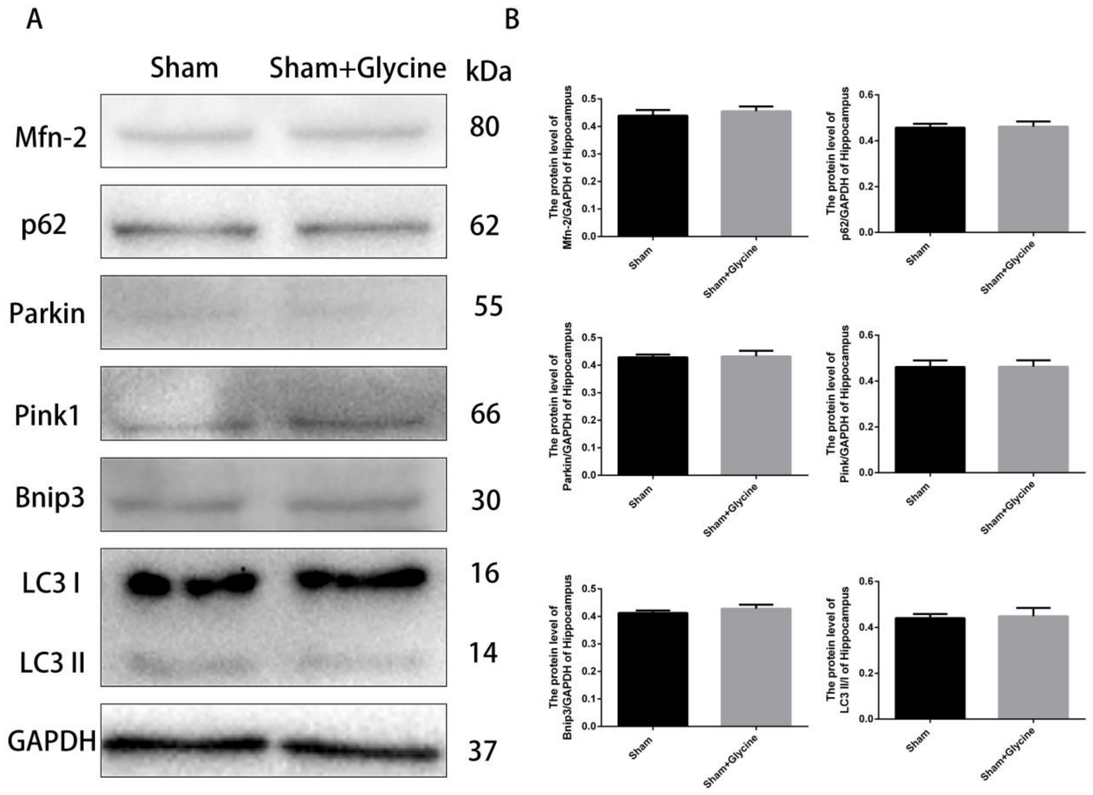

Suppl figure 2. The dosage of glycine pretreatment has no effect on normal hippocampus status of neontal rats.

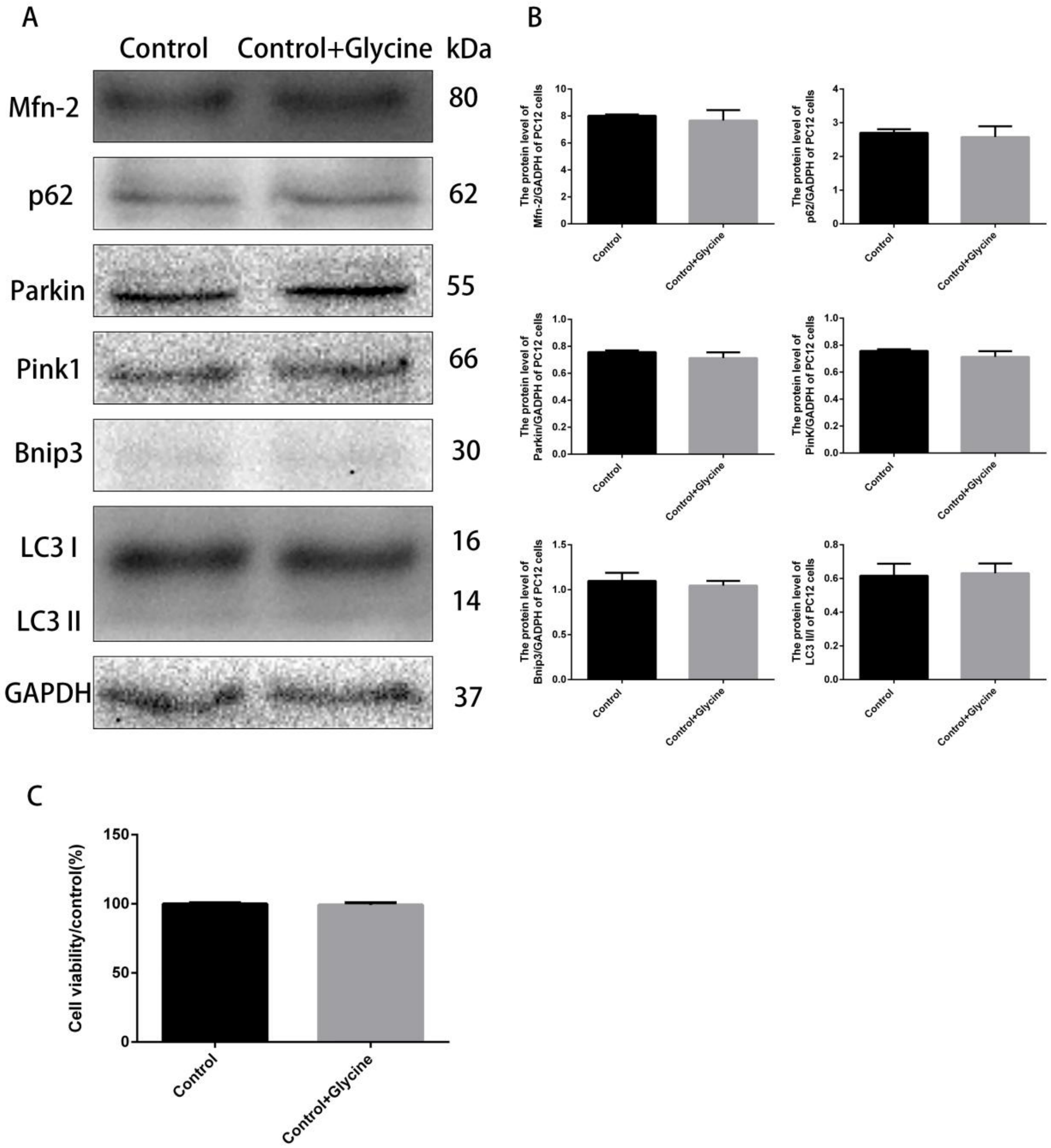

Suppl figure3. The dosage of glycine administration has no influence on normal status of PC12 cells.
